# Responsive feedback: Towards a new paradigm to enhance intervention effectiveness

**DOI:** 10.12688/gatesopenres.12937.2

**Published:** 2019-05-28

**Authors:** K. Viswanath, Christina Synowiec, Sohail Agha

**Affiliations:** 1 Harvard T. H. Chan School of Public Health (HSPH), Dana-Farber Cancer Institute (DFCI), Boston, MA, 02215-5450, USA; 2 Results for Development, Washington, DC, 20036, USA; 3 Global Development, Bill & Melinda Gates Foundation, Seattle, WA, 98109, USA

**Keywords:** Implementation science, responsive feedback, feedback loops, adaptive implementation, theory of change, monitoring and evaluation

## Abstract

The current dominant models of intervention design in the development sector do not account for the complexity and unpredictability of implementation challenges. Decision makers and implementers need timely feedback during implementation to respond to field realities and to course-correct. This letter calls for a new approach of “responsive feedback” or “feedback loops” that promotes interactions between project designers, implementers, researchers and decision-makers to enable course corrections needed to achieve intended outcomes. A responsive feedback approach, in theory, should be agile, flexible, adaptive, iterative, and actionable. There can be multiple challenges associated with incorporating this approach into practice including donor requirements, organizational structure and culture, concerns about the additional time required to adopt such an approach, resource and operational constraints, the absence of skill sets needed for such an approach within smaller organizations and inadequate inter-departmental communication. However, these barriers to adaptation can be overcome. For responsive feedback to become a part of the culture of development organizations, commitment is needed from donors, decision-makers, project designers and implementers. We believe that, to generate opportunities for learning and adaptation, donors should provide the stimulus to break down silos between implementers and researchers.

The current dominant models of intervention design are fraught with challenges that make it difficult for programs to be responsive to the complexity and unpredictability of implementation challenges. Partly, the dominant model is influenced by medical or pharmaceutical trial models which use randomized controlled trials as the gold standard, assume high degree of fidelity and certainty to intervention and seemingly invariant conditions when the stimulus or drug is administered. The reality of social and behavioral interventions, on the other hand, are far too different posing tremendous challenges. For example, current tools and models for understanding deviations from what was originally expected and what is observed during implementation do not allow programs to respond with the flexibility and agility required in rapidly changing field situations. Limited flexibility in programmatic response is often seen when limited or no feedback is provided to decision-makers, including implementers, during the implementation process. This lack of agility may be a function of the types of data, study designs and management skills needed to respond to changing implementation needs. It is equally possible that an organization’s culture may not be open to receiving and acting on feedback.

A culture of continuous learning may be needed for a host of reasons, including changing consumer expectations, unexpected implementation constraints or faulty assumptions made at the project design stage. Our conventional approach to assessing the effectiveness of interventions by collecting data at the end of the intervention, provides limited opportunity to be responsive to day-to-day developments in the field. The long interval between the end of the intervention and completion of the evaluation means that the evidence generated is of limited utility in course correction and in providing timely feedback to implementers. Moreover, the lack of a systematic process for linking ongoing implementation learnings to modifications in project design - the feedback loop - often precludes adaptive implementation.

Recent thinking has posed a bold challenge to this orthodoxy aided by developments in theory, methods and practice, calling for an approach that promotes interaction between project designers, implementers, researchers and decision-makers, to encourage adaptation through learning. This newer approach, often using such terms as “responsive feedback” or “feedback loops,” calls for timely assessments that provide actionable feedback to implementers to course correct and achieve intended outcomes. Three developments have contributed to the increasing momentum of this approach. One, the advent of information and communication technologies (ICTs) has increased opportunities to collect, analyze and disseminate evidence/data more rapidly. Two, multi-disciplinary and multi-sector thinking has allowed for greater sharing, adoption and adaptation of lessons from different sectors and is increasingly infusing thinking in the development sector. Three, there is a shift among implementing organizations from considering monitoring and evaluation as an accounting or “auditing” function ( Colquhoun *et al*., 2017) to a learning function that drives continuous improvement.

In this letter, we are calling for a wider adoption of this new approach to enhance the effectiveness of interventions, ultimately leading to improvements in the lives of people for whom the interventions are intended. We will briefly touch upon some important issues regarding challenges in adopting a responsive feedback approach, and ways to overcome these challenges. This is not meant to be an exhaustive review of a responsive feedback approach as much as an effort to generate dialogue among donors and practitioners among others. Our approach builds on earlier work in social and behavioral sciences that offered multiple frameworks for implementation and from literature on management that especially integrated implementation sciences with improvement sciences ( Balasubramanian *et al*., 2015; Gaglio *et al*., 2013; Glanz *et al*., 2015; Green & Kreuter, 2005). We must also note that the RFM approach builds on and is complementary to similar dialogues in the development sector such as “Doing Development Differently” which also call for “rapid cycles of planning, action, reflection and revision” in implementing social change ( Harvard Center for International Development, 2014).

## How to label the new approach

To start with, one challenge worth noting is that there is a lack of consensus over terms and definition of what responsive feedback is. While developing a consensus is not the aim of this letter, we contend that it is critical to develop standard terminology and a broadly acceptable definition of what this approach may be called. We argue for the terms “responsive” and “feedback” to characterize the philosophy and goals of this approach. A responsive feedback approach reduces the tension between traditional monitoring and evaluation and traditional implementation and decision-making functions. It proposes that the design of research activities should be driven by the explicit intention of providing actionable insights to implementers. For the learning function to be effective (as judged by whether it leads to improved implementation and design decisions), interaction among researchers, implementers, and other decision-makers is important at defined moments throughout the project’s life cycle. In turn, the learning questions and the timing of these moments should be guided by the project’s Theory of Change.

## Theories of Change and responsive feedback approach

A Theory of Change (ToC) is critical as, by identifying pathways and markers of course-correction, it can inform factors that influence the effectiveness of an intervention to ensure that short-term to long-term objectives are met. However, few interventions have an explicit ToC and hypotheses that are continuously tested and refined to improve the interventions. We argue that a ToC is an essential complement of a responsive feedback approach.

A ToC approach is about making our forward-thinking narrative explicit – and the assumptions that underlie one’s thinking. It clarifies how we see cause-effect relations between activities or actions and their intended changes, ensures that causal links and assumptions behind the links are explicit, and helps hone in on the relationship between activities and the achievement of long-term goals. Instead of becoming fixated on what the program is currently doing, it draws people’s minds to the activities that are needed to achieve the goals. This leads to better planning in that activities are linked to a detailed understanding of how change actually unfolds.

ToCs are largely focused on uncovering and critically appraising assumptions, with learning as a key goal of the process. Responsive Feedback Mechanisms (RFM) are a tool to support the practices of learning and adaptive thinking – making ToCs and RFM critical complements to one another. And, by using a collaborative and participatory process, developing a ToC should involve discussion about existing understandings of how change should happen and articulating the underlying assumptions. Through this process one may identify where there are structural inconsistencies or contradictions, particularly around cause-effect relations in the logic of change pathways, or where there is uncertainty, pinpointing where RFM can be most useful.

In summary, these points identify potentially powerful use of ToCs and RFM to drive program improvement – with the ToC setting the learning agenda and uncovering assumptions in need of validation, and RFM providing the tools and methods to do so.

## Characteristics of RFM

What are some of the principal characteristics of responsive feedback approach and the interventions informed by RFM? There are five key features:

1) *Agility and flexibility*: RFM designs should be agile and flexible enough to capture changes (or lack of changes) due to the intervention.
2) *Adaptive*: RFM-driven interventions are not fixed but are adaptive to feedback based on context and situation.
3) *Iterative*: The culture of experimentation suggests an openness to test and change the intervention in response to the latest insights, often building in multiple rounds of feedback loops throughout implementation.
4) *Responsive*: The RFM approach should be sensitive to the needs of implementers and decision-makers at each stage of the intervention, driven by methods that take a problem-driven approach to answering key learning questions.
5) *Actionable*: The data generated through the RFM approach are relevant and timely to inform key design and implementation questions.

## Challenges in implementing a responsive feedback culture

While the idea of responsive feedback is slowly being appreciated, it is by no means without its challenges, particularly in its operationalization. These challenges include the organizational structure and culture, capabilities of both researchers and implementers and resource constraints.

(a) *Organizational culture*: The culture of an organization has profound implications for whether RFM can be successfully executed by the organization implementing the intervention. One, the leadership of the organization should be open to the philosophy of experimentation and iterative improvement including the presence of champions who can advocate for RFM. Second, any silos between departments such as Monitoring, Learning and Evaluation (MLE) and program design and implementation will have to break down to facilitate communication and coordination. Third, and this is linked to the ToC, there may be a reluctance to question the assumptions built into program design resulting in an unwillingness to acknowledge preconceived notions even in the face of contrary evidence.
(b) *Program design*: Implementation timelines, resource and operational constraints, or reporting compliance restrictions may not allow for the flexibility needed to allow for iterative design.
(c) *Organizational structure:* This includes such characteristics as the size and complexity of organizations. Large organizations, in theory, have personnel with specialized functions and separate departments for program design, grant writing, implementation, MLE, frontline workers, information technology (IT) support etc. While large organizations may have the personnel and structure to perform such specialized functions, it may be difficult to change a culture that deters responsiveness of the MLE group to the needs of the designers and implementers with timely feedback. On the other hand, while the division of labor is likely to be simpler in smaller organizations, they may lack the specialization, bandwidth and resources to adopt RFM effectively.
(d) *Human capital*: It is vital that personnel are trained with appropriate skills in adopting and implementing RFM. This requires specialized skills such research design, programming skills, IT support, critical appraisal and utilization of evidence and execution. One solution is to promote “task shifting” where one can invest in human capital for some of the technical tasks such as evidence-interpretation and program adaptation and shift these tasks from highly technical personnel to decision-makers and implementers within the same organization ( Fulton *et al*., 2011). Digital technologies can be particularly helpful in reaching and training health workers and providing the necessary skills for continuous learning ( NAS, 2017).
(e) Most organizations are *resource-challenged* with multiple demands for their limited resources. Organizations may prefer setting aside a bulk of the money for intervention design and implementation rather than monitoring and evaluation. This is particularly important for organizations that look at MLE and ensuing feedback as a “luxury” available only when programmatic needs have been met. However, “adaptive management,” a culture and structure that allows for experimentation, testing and iterative learning may be able to minimize the adverse impact of limited resources by distributing the responsibility of learning and adaptation across a broader range of actors within the same organization.
(f) *Funding*: Funding and funders influences the level of flexibility in design and implementation. Implementers could be penalized for “experimentation” or “iterating” with program design. And funder reporting requirements may prioritize basic monitoring activities without encouraging ongoing adaptation and learning throughout implementation, putting RFM at odds with other priorities.
(g) Inter-organizational coordination: Implementation of most social and behavioral interventions takes place under complex societal conditions and that includes multiple organizations and stakeholders with varying degree of expertise, experiences, resources and histories. The “network” effect of complexity when multiple stakeholders are involved call for greater understanding, collaboration and coordination in implementing RFM-informed interventions.

## Opportunities

While there are challenges in adopting responsive approaches, there are a number of ongoing developments that make it conducive to accelerate their adoption.

One among them is the alacrity with which the social sector has adopted the tools stemming from the revolution in information and communication technologies. We are witnessing the emergence of the idea of digital health, “a set of activities and tools that encompass health, information and communication technology (ICT), including mobile health (mHealth), health information technology (IT), health information systems, wearable devices, telehealth, and telemedicine” ( National Academy of Sciences, 2017). Development organizations are increasingly taking advantage of these changes particularly as barriers to adaption are lowering.

But perhaps more foundational is the shift among development practitioners to participatory, actionable, and adaptive MLE approaches. This cultural shift is accompanied by an expansion in the ways traditional monitoring and evaluation tools are being applied to support ongoing learning opportunities – by building in participation of key stakeholders throughout design and implementation or by supporting capacity development for organizational staff on MLE.

## Implications

In this letter, we do not make any claims about how easy it is to adopt an RFM approach nor do we discuss in any depth the modes of observation for data collection to facilitate decision-making. Program planners and managers have to prioritize “what” to observe and “when” to observe based on the ToC to help in decision making based on such considerations as costs, order of importance, skills and practicality in decision making ( Hornik, 1992).

 A culture of responsive feedback begs commitment from all parties involved in project design, implementation and learning. Donors need to provide the impetus for interactions between implementers and researchers that result in opportunities for learning and adaptation. This may require an upfront commitment of time and resources to test areas of uncertainty within the ToC using formative research or small experiments. It may have implications for what functions are needed for project management, for the type of reporting needed and possibly even for what is admissible as learning. Requests for proposals would have to highlight the importance of returning to the ToC periodically and using it as a way of navigating improvements in implementation. At the same time, commitments would be needed of implementers, decision-makers, researchers, and other development practitioners to embrace the RFM approach, actively participate in continuous learning, and act on the evidence generated. Our organizations have each experimented in different ways with using RFM to enhance intervention effectiveness and believe in its power to effect development change on the pathway to impact.

## Disclaimer

The views expressed in this article are those of the authors. Publication in Gates Open Research does not imply endorsement by the Bill & Melinda Gates Foundation.

## Data and software availability

No data are associated with this article.
