# Supplementary material for: Responsive feedback: Towards a new paradigm to enhance intervention effectiveness
Source: Gates Open Res. 2019 May 28;3:781. Originally published 2019 Mar 8. [Version 2] doi: 10.12688/gatesopenres.12937.2 (PMC6480401; doi:10.12688/gatesopenres.12937.2)
Supplement: Peer review history

# Peer review: Reviewer response for version 1

**DOI:** 10.21956/gatesopenres.14040.r26982

**Published:** 2019-04-04

**Authors:** Robert C. Hornik

**Affiliations:** 1 Annenberg School for Communication, University of Pennsylvania, Philadelphia, PA, USA

This is a thoughtful, well written letter making the case for building stronger feedback mechanisms into implementation of programs. The authors argue for the benefits of moving beyond the narrow use of research in many current projects, emphasize the essential role of an explicit theory of change, and summarize some of the barriers to building in responsive feedback mechanisms. They charge donors with the responsibility for overcoming such barriers.  

 I bring a particular perspective to this discussion, a long term engagement with the use of mediated communication within development and particularly health-related interventions. That experience has led me to a perspective that overlaps with the authors' framework, but may also differ from it in some details.  

 The hardest problem in moving towards a ‘responsive feedback’ culture is the transformation of organizational culture in practice. There are a very large number of research-able questions that can be asked; determining which ones have priority, framing potential action choices concretely so that evidence can inform those decisions, establishing what quality of evidence will be adequate to underpin decisions, and being ready to respond when the feedback comes in are all part of this transformation. The argument for the responsive feedback culture in the abstract is meritorious; implementing it within a real intervention may be a struggle. 

 As an example, here is a startup list of questions a mass media-based anti-tobacco program might monitor: whether pretesting has established the promise of the arguments to be emphasized in messages; whether the media channels actually transmitted messages on schedule; whether their primary audiences recall hearing major messages; whether those audiences have been persuaded by the arguments contained in the messages (e.g. that tobacco use is addictive); whether they have intended to change behavior and whether they have actually changed behavior. Monitoring can assess whether responses to these outcomes have been universal or differentiated by characteristics of the audience. Monitoring can ask whether the media messages generated social discussion and diffusion through interpersonal channels, or whether institutional actors were influenced as well and made concrete policy changes (e.g. banning tobacco use in bars).

 This already long list of potential monitoring questions, which would be driven by the theory of change articulated as the basis for the program, can be multiplied. But even this restricted list has to be prioritized reflecting the cost to do research, the skills available, the leverage an answer might have for program decisions (Hornik, 1992 ^1^). Then questions have to be formulated in ways that allow obtained answers to drive intervention decisions; will high quality samples and closed-ended face-to-face interviews be required, or will intercept interviews or focus groups or surveilled social media discussions provide adequate information? Will randomized controlled experiments be needed or will naturalistic observational studies be sufficient? Then, what action would be taken if, for example, 40% of the audience recalled exposure to the messages, or 15% reported sharing the messages with others? Is there enough flexibility in the budget and is there the required lead time to make potential needed changes? One can easily imagine program managers being exhausted merely by the process of articulating questions. How to implement a responsive feedback system across the wide range of programs and individuals charged with doing so, is the hard question. The answer may be that some feedback is better than none, and even programs who will be constrained in what they can realize can exploit some such feedback.

 A second thought stimulated by the letter leads me to differentiate three quite different forms of responsive feedback that might be incorporated, including monitoring, support for causal claims, and operational testing. All of the example questions above fall under monitoring, the effort to assess whether things are changing consistently with an articulated theory of change. My own guess is that monitoring is where most actionable research will fall. But monitoring may not permit confident causal claims; monitoring may show that the audience increasingly believes that tobacco use is addictive, but that may not allow a claim that the campaign affected that belief if other forces also might be influencing the belief. A more elaborate research approach may be required. Similarly monitoring may establish that ads are inadequately recalled; it will not establish how to make sure they are better recalled—operational testing (comparing proposed A and B strategies for increasing exposure) may be required. 

 In sum, I very much support the argument in this letter. Turning it from sensible abstract argument to implementable program will be hard. In particular doing such implementation adapted to the realistic context and circumstances of programs that will vary in size, in skills of staff, and of research resources will be demanding. Making sure that the data gathering does not become an end in itself, as is too often the case with program research, but actually serves the evolution of programs will be a constant struggle. It will be worth doing.

I have read this submission. I believe that I have an appropriate level of expertise to confirm that it is of an acceptable scientific standard.

## References

[1] HornikR: Social Science Research to Assist and Assess AIDS Prevention Programs.1992; 10.1007/978-3-0348-7211-9_2 17-31 10.1007/978-3-0348-7211-9_2

# Response

**Published:** 2019-04-24

**Authors:** Sohail Agha

In this letter, we do not make any claims about how easy it is to adopt an RFM approach nor do we discuss in any depth the modes of observation for data collection to facilitate decision-making. Program planners and managers have to prioritize “what” to observe and “when” to observe based on the ToC to help in decision making based on such considerations as costs, order of importance, skills and practicality in decision making (Hornik, 1992).

# Peer review: Reviewer response for version 1

**DOI:** 10.21956/gatesopenres.14040.r26981

**Published:** 2019-03-25

**Authors:** Steven Chapman

**Affiliations:** 1 IKEA Foundation, Leiden, The Netherlands

I am grateful to the authors for publishing these remarks from the 2 ^nd^ Summit of the Curve Initiative, and for encouraging donors to commit to creating the conditions for Responsive Feedback Mechanisms, particularly ‘an upfront commitment of time and resources to test areas of uncertainty within the ToC using formative research or small experiments.’ I recommend approving this for indexing as is because of the importance of the substance of the letter and the opportunity that open peer review gives to comment. I agree with the overall argument put forth here in terms of what RFM needs to be, challenges in implementing it, and the opportunities described.

 The authors begin by stating that it is critical to develop standard terminology and a broadly acceptable definition of what this approach may be called and then argue for a new term of ‘Responsive Feedback Mechanisms.’ A new term may indeed be needed, but I wanted to be convinced given the now five years of experience behind ‘Doing Development Differently’ and nearly ten years behind ‘Developmental Evaluation’. Doing Development Differently has a manifesto in which the blending of design and implementation through rapid cycles of planning, action, reflection, and revision to foster learning from both success and failure is one of six principles. Developmental Evaluation supports innovation development to guide adaptation to emergent and dynamic realities in complex environments. These initiatives appear to overlap at least in part with RFM and that merits analysis at some stage. 

 The authors put Theories of Change as essential to responsive feedback approaches, and describe well their use and promise in intervention planning, monitoring and evaluation. I would welcome comment from the authors about how to think about the proper scope of the ToC, particularly when there is an absence of evidence on a critical success factor. When that is the case, should the intervention be broken up into two parts, one to build evidence about the relationship of the intervention to the critical success factor? Or should it be kept together to learn within the context of a complex intervention?

 Lastly, the authors describe many organizational challenges to implementing a RFM, from culture, to structure, human capital, and resource constraints. The authors appear to limit their comments to challenges within a single organization, but many complex interventions are implemented by multiple organizations, increasing these challenges significantly. I would welcome comment from the authors on what learning there now is about how to reduce these challenges.

I have read this submission. I believe that I have an appropriate level of expertise to confirm that it is of an acceptable scientific standard.

# Response

**Published:** 2019-04-24

**Authors:** Sohail Agha

We thank the reviewer for pointing out similar dialogues in other sectors. We added the relevant citation and referred to the specific movement of Doing Development Differently.

Clearly, this involves a judgement call within the context of the specific intervention and there is a not a lot of experience yet on how best to proceed in such an instance. To the extent possible, however, we feel that the project should build evidence about the relationship of the intervention to the critical success factor if such evidence is not available.

We agree with the reviewer that interventions are implemented in a complex environment involving multiple organizations. We added this to our list of challenges.

# Peer review: Reviewer response for version 1

**DOI:** 10.21956/gatesopenres.14040.r26980

**Published:** 2019-03-19

**Authors:** W. Douglas Evans

**Affiliations:** 1 Department of Global Health, The George Washington University, Washington, DC, USA; 2 Department of Prevention and Community Health, Milken Institute School of Public Health, The George Washington University, Washington, D.C., USA

This is an interesting and well-written article on the importance of a new approach to using responsive feedback or feedback loops to program design, implementation, and evaluation. It makes a valuable contribution to the literature and to this reviewer follows in the tradition of related theoretical frameworks, including RE-AIM (Glasgow) and PRECEDE-PROCEED (Green & Kreuter). At the same time it goes beyond these traditional and largely theoretical models.

 The new responsive feedback approach offers practical recommendations and an implementation and monitoring and evaluating model for future programs. As such, it goes beyond traditional theories. However, I believe the article could be strengthened in two respects:

1. First, there should be additional citations to related preceding theories, such as those noted above. These should be acknowledged when the topics of feedback loops and their role in customer orientation and satisfaction are discussed. This section should perhaps also acknowledge the business and management sciences literature on related topics on continuous process improvement and cite some relevant literature (e.g. Drucker, 2014 ^1^).
2. Second, the article would really benefit from at least one specific example of a feedback loop in action in a relevant programmatic context. The discussion is a bit abstract and a concrete example, even if just described in 2-3 sentences with a citation, would be very helpful to readers.

Overall, really nice article. I look forward to seeing this approach applied and studied more broadly in the development sector and related fields.

I have read this submission. I believe that I have an appropriate level of expertise to confirm that it is of an acceptable scientific standard.

## References

[1] DruckerP: Innovation and Entrepreneurship.2014; 10.4324/9781315747453 10.4324/9781315747453

# Response

**Published:** 2019-04-24

**Authors:** Sohail Agha

We referred to earlier work in this area and suggested how our work builds on these earlier writings. We added the relevant citations.

 As part of the work under the Curve CoP, we are in the process of developing 4 concrete examples of feedback loops.

# Peer review: Reviewer response for version 1

**DOI:** 10.21956/gatesopenres.14040.r26984

**Published:** 2019-03-11

**Authors:** Richard L. Wright

**Affiliations:** 1 Unilever PLC, Port Sunlight, UK

This letter makes an important point concerning the development sector's approach to the implementation of (behavioral) interventions. There is a pressing need for many projects to build in room to course correct; workers must also adapt the appropriate mind-set and we must ensure that the appropriate skills and tools are available.

 While I agree with the article, I do think that it could be clearer in describing 'The current dominant models of intervention design'. The authors assume that these are known to all readers and that there is a common understanding of how these models drive intervention design and implementation.  

 My own belief (without wishing to put words into the mouths of the authors) is that models of intervention design are borrowed from pharmaceutical human trials and applied to behavioral science (rather like RCTs). Within pharmaceutical human trials there is a high degree of certainty around the intervention design - the drug in question having gone through multiple early tests. Behavioral science is different - we start out with humans and high degrees of uncertainty. Therefore, there is a strong need to be adaptive in the early stages and this (as the article points out) needs to be recognised.  

 The authors may disagree with my analysis, however I still think they need to describe the dominant models in more detail, so the reader is clear on what they are arguing against.

I have read this submission. I believe that I have an appropriate level of expertise to confirm that it is of an acceptable scientific standard.

# Response

**Published:** 2019-04-24

**Authors:** Sohail Agha

The reviewer raises an important issue and we agree that our social and behavioral intervention designs and evaluation have been considerably informed if not influenced by designs of drug trails. The focus on RCTs as a gold standard is a particularly telling illustration of this argument. We incorporate this point at the very beginning of the essay.
